# Effect of insulin or insulin-like growth factor-I administration at mid-luteal phase of the estrous cycle during superovulation on hormonal profile of Sahiwal cows

**DOI:** 10.14202/vetworld.2018.1736-1741

**Published:** 2018-12-27

**Authors:** S. K. Sheetal, Shiv Prasad, H. P. Gupta

**Affiliations:** Department of Veterinary Gynaecology and Obstetrics, College of Veterinary and Animal Sciences, Govind Ballabh Pant University of Agriculture and Technology, Pantnagar, Uttarakhand, India

**Keywords:** insulin-like growth factor-1, insulin, progesterone, Sahiwal, superovulation

## Abstract

**Aim:**

The present study was designed to study the effect of insulin and insulin-like growth factor-1 (IGF-I) administration during luteal phase of the estrous cycle on the hormonal profile of Sahiwal embryo donor cows during superovulation.

**Materials and Methods:**

Cows (n=18) were selected and divided into three groups; control (n=6, untreated), T-I (n=6, insulin-treated), and T-II (n=6, IGF-I treated). Insulin and IGF-I were given S/C on 5^th^, 6^th^, 7^th^, and 8^th^ days of estrous cycle. Superovulatory treatment was started on day 9^th^ of the estrous cycle. With the sixth dose of follicle-stimulating hormone, prostaglandin was injected to induce superovulatory heat. The superovulated cows were bred, and superovulatory response of each animal was recorded. The embryos were collected non-surgically on the 7^th^ day of superovulatory estrus. About 15 ml blood without anticoagulant was collected on days 5, 7, 9, 11, 13, 15, 17, 19, and 21 or day of embryo recovery where day 0 of estrous cycle was taken as day of estrus. Serum was separated, centrifuged, and transferred into sterilized serum vials. All samples were stored at −20°C till analysis. Progesterone, insulin, and IGF-I were estimated in blood serum by radioimmunoassay using radioimmunoassay kits.

**Results and Discussion:**

The mean concentration of progesterone on days 7 and 11, insulin on days 7 and 9, and IGF-I on days 5, 7, 9, 11, and 15 was significantly higher in insulin-treated T-I groups as compared to untreated control.

**Conclusion:**

It may be concluded that exogenous insulin administration during mid-luteal phase may be helpful in follicular and embryonic development through modulation of progesterone, insulin, and IGF-I in indigenous (*Bos indicus*) Sahiwal embryo donor cows.

## Introduction

Bovine embryo transfer technology is being widely used around the world for the production of breeding stock. The semen produced from such elite bulls is disseminated for livestock improvement. In this technique, genetic contribution of both the male and female is utilized simultaneously leading to faster genetic improvement. In India and other Asian countries, the demand to multiply the genetic material of valuable *Bos indicus* females has increased. Sahiwal cow is one of them which is more suitable to our climatic conditions as compared to *Bos taurus*. However, poor superovulatory response, inappropriate storage, and higher embryo mortality leading to lower conception rate in recipient are some of the major problems that limit field application in large scale.

Insulin application to modulate the reproduction is a recent development. *In vitro* studies revealed insulin and insulin-like growth factor-1 (IGF-I) as important mediators of follicular development, steroidogenesis, oocyte maturation, and subsequent embryo development [1]. Administration of insulin increases intrafollicular and peripheral IGF-I levels in cattle [2] and positively correlated with the peripheral concentration of insulin and IGF-I [3]. Hence, the use of insulin may be considered as an alternative approach to improve superovulatory response.

IGF-1 of endocrine origin has been positively linked to the reproductive performance of cow [4,5] and ewes [6]. Future fertility of cows [7] and the timing of first and postpartum ovulations in dairy cows [8] can be predicted by measuring the circulating IGF-1 level. However, the efficacy of IGF-1 administration and their level in the blood to forecast the success of MOET/SOET programs in cattle is poor. These metabolic hormones influence the superovulatory response by increasing the population of gonadotrophin-dependent follicles.

Thus, the present work was designed to study the effect of insulin and IGF-I treatment on alteration in hormonal profile in embryo donor Sahiwal cows.

## Materials and Methods

### Ethical approval

The present investigation was carried out after the approval of the Institutional Animal Ethics Committee.

### Study design

It was designed to study the effect of insulin and IGF-I administration during the mid-luteal phase of the estrous cycle on the hormonal profile of Sahiwal donor cows. For embryo donor, Sahiwal cows (n=18) were selected and divided into three groups; control (n=6, untreated), T-I (n=6, insulin-treated), and T-II (n=6, IGF-I treated). The sample size was only 18 because superior qualities of cows were selected based on per-rectal examination of herd. Further, the cost of superovulation and embryo transfer is very high due to the higher price of follicle-stimulating hormone (FSH) (Folltropin-V), which limits our sample size. However, this sample size was enough for statistical analysis. Insulin was given at 0.25 IU/kg body weight by S/C to the animals of the treatment Group I, on 5^th^, 6^th^, 7^th^, and 8^th^ days of the estrous cycle. IGF-I was given at 10 µg total dose per day by S/C to the animals of the treatment Group II, on 5^th^, 6^th^, 7^th^, and 8^th^ days of estrous cycle. Superovulatory treatment was started on day 9^th^ after the onset of standing estrus (Folltropin-V, Vetoquinol, Canada, 30 mg twice daily at 12 h intervals for 4 days, and total dose 240 mg in 8 divided doses). With the sixth dose of FSH, prostaglandin (PGF) was injected to induce superovulatory heat. The superovulated cows were bred 2 times at 12 h interval through artificial insemination using good quality frozen semen, and superovulatory response of each animal was recorded based on a total number of corpus luteum (CL) and unovulatory follicles present, with the help of per-rectal examination and sonography of both ovaries. The embryos were collected non-surgically on the 7^th^ day of superovulatory estrus. About 15 ml blood without anticoagulant was collected on days 5, 7, 9, 11, 13, 15, 17, 19, and 21 where day 0 of estrous cycle was taken as day of estrus. The days 9, 11, 13, and 21 correspond to day of the initiation of FSH treatment, day of PGF2α injection, day of superovulatory estrus, and day of embryo recovery, respectively. The sterilized test tube kept at room temperature as a slant for 1 h for separation of blood serum. Serum was separated, centrifuged at 3000 rpm for 15 min, and transferred into sterilized serum vials. All samples were stored at −20°C until analysis.

### Hormonal estimation

Progesterone, insulin, and IGF-I were estimated in blood serum by radioimmunoassay (RIA) using RIA kits (M/S Beckman Coulter IM 1188). The progesterone estimation (analytical sensitivity: 0.03 ng/mL or 0.10 nmol/L) in plasma samples was done using progesterone C.T. RIA kit. The insulin estimation (analytical sensitivity: 0.49 µ IU/mL) in serum samples was done using insulin(e) IRMA kit. The IGF-I estimation (Analytical sensitivity: 4.55 ng/mL) in serum samples was done using IGF-I IRMA kit at IVRI (Nuclear Research Laboratory under Division of Physiology and Climatology).

### Statistical analysis

The data were analyzed statistically using analysis of variance [9]. The analysis of data was applied both within the group and between the groups for the concentrations of progesterone, insulin and insulin like growth factor-I (IGF-I). Differences were considered significant at p<0.05 and p<0.01.

## Results

### Progesterone

**Figure-1:**
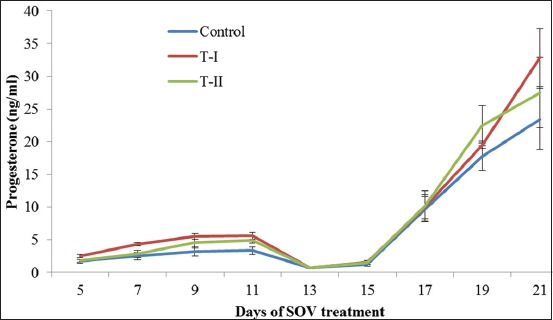
Mean (±standard error) serum progesterone concentration (ng/ml) of different groups on different days of estrous cycle/superovulatory treatment of Sahiwal donors.

The mean serum progesterone concentration at different phases of the estrous cycle in control, T-I (insulin-treated), and T-II (IGF-I treated) groups have been presented in Table-1 and Figure-1. Its concentration on day 5^th^ of estrous cycle did not differ significantly among the groups. On day 7, there is a significant difference found between control and T-I (insulin-treated) groups. Further, its concentration on the 9^th^ day, that is, day of initiation of FSH treatment differs non-significantly (p>0.05) but higher in T-I and T-II than the control group. The levels of progesterone remained constant at day 9^th^ and 11^th^ as functional CL were present on ovary of each cow. The concentration of serum progesterone declined significantly following PGF2α injection in all the groups and reached its basal level on the day of superovulatory estrus (<1 ng/ml) in all the groups. Its concentration increased in all groups following superovulatory estrous, that is, from 13^th^ day to 21^st^ or day of embryo recovery. The peak value of progesterone reached on the day of embryo recovery, and the highest value was found in T-I group.

**Table-1:** Serum progesterone concentration (ng/ml) of different groups on different days of estrous cycle/superovulatory treatment of Sahiwal donors.

Days of estrous cycle	Groups		

	Control (n=6)	T-I (n=6, insulin at 0.25 IU/kg b. wt. S/C)	T-II (n=6, IGF-I at 10 µg total dose per day S/C)
Day 5	1.73±0.29^ba^	2.54±0.23^ba^	1.80±0.23^a^
Day 7	2.49±0.59^baA^	4.36±0.25^baB^	2.85±0.55^aBA^
Day 9	3.24±0.69^ba^	5.55±0.41^ba^	4.60±0.84^a^
Day 11	3.35±0.58^baA^	5.62±0.52^baB^	4.98±0.49^aBA^
Day 13	0.69±0.04^a^	0.71±0.07^a^	0.76±0.07^a^
Day 15	1.22±0.28^ba^	1.55±0.30^a^	1.51±0.29^a^
Day 17	9.71±1.93^cb^	10.05±1.85^b^	10.21±2.34^a^
Day 19	17.73±2.09^dc^	19.50±0.58^c^	22.46±3.03^b^
Day 21 or DER	23.46±4.70^d^	32.85±4.45^d^	27.50±5.40^b^

(Means bearing different superscripts differed significantly (p<0.05) within the groups (a, b, c, d) and between the groups (A, B). IGF-I=Insulin-like growth factor-I

### Insulin

**Figure-2:**
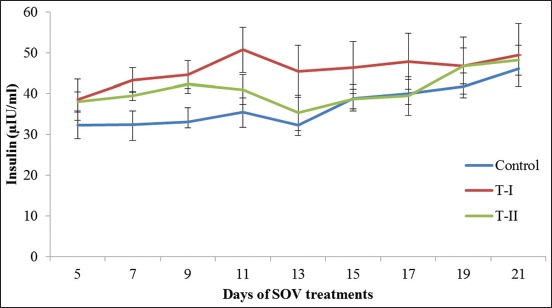
Mean (±standard error) serum insulin concentration (µ IU/ml) of different groups on different days of estrous cycle/superovulatory treatment of Sahiwal donors.

The mean serum insulin concentration during different phases of the estrous cycle in control, T-I (insulin-treated), and T-II (IGF-I treated) groups have been presented in Table-2 and Figure-2. Its concentration on days 7 and 9 differs significantly (p<0.05) between control and T-I groups. The concentration of insulin on the 9^th^ day, that is, day of initiation of FSH treatment was significantly (p<0.05) higher in T-I than the control group (Table-2). Its concentration further increased on the 11^th^ day in control and T-I groups but increase over a 9^th^ day in these groups was non-significant. The mean insulin concentration started declining following PGF2α injection on 11^th^ day in all the three groups and reached lowest on the day 13 i.e., day of superovulatory estrus and differed non-significantly within groups (p>0.05). The mean insulin concentration further increased in all groups from 13^th^ day to 21^st^ or day of embryo recovery (DER). The peak value of insulin was recorded on the 21^st^ day or DER in all groups and differs significantly from 13^th^ day in control, and the highest value was found in T-I group.

**Table-2:** Serum insulin concentration (µ IU/ml) of different groups on different days of estrous cycle/superovulatory treatment of Sahiwal donors.

Days of the estrous cycle	Groups		

	Control (n=6)	T-I (n=6, insulin at 0.25 IU/kg b. wt. S/C)	T-II (n=6, IGF-I at 10 µg total dose per day S/C)
Day 5	32.32±3.41^a^	38.53±5.04	37.94±2.51
Day 7	32.38±3.85^aA^	43.31±3.04^B^	39.43±1.17^BA^
Day 9	33.12±1.54^baA^	44.63±3.46^B^	42.37±2.43^BA^
Day 11	35.51±3.77^ba^	50.75±5.57	41.00±3.68
Day 13	32.23±2.54^a^	45.48±6.39	35.29±4.37
Day 15	38.81±3.1^ba^	46.41±6.37	38.71±2.4
Day 17	40.01±2.62^ba^	47.92±6.83	39.44±4.73
Day 19	41.69±2.69^ba^	46.82±7.00	46.77±4.38
Day 21 or DER	46.11±1.57^b^	49.42±7.71	48.22±3.68

Means bearing different superscripts differed significantly (p<0.05) within the groups (a, b, c, d) and between the groups (A, B). IGF=Insulin-like growth factor-1

### IGF-I

**Figure-3:**
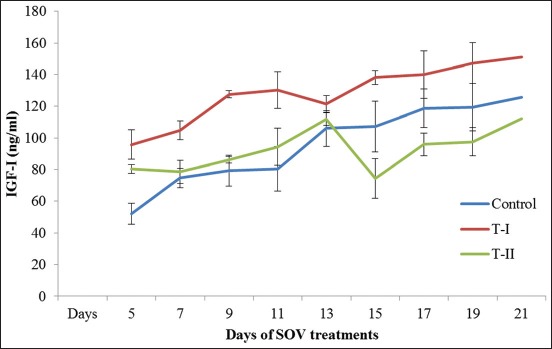
Mean (±standard error) serum insulin-like growth factor-1 concentration (ng/ml) of different groups on different days of estrous cycle/superovulatory treatment of Sahiwal donors.

The mean serum IGF-I concentration at different phases of the estrous cycle in control, T-I (insulin-treated), and T-II (IGF-I treated) groups have been presented in Table-3 and Figure-3. Its concentration on day 5^th^ differs significantly (p<0.05) in T-I and T-II groups from control, on 7^th^, 9^th^, and 11^th^ days T-I from control and T-II, on 15^th^ day significant from each other, and on 19^th^ day between T-I and T-II (p<0.05). The concentration of IGF-I on the 9^th^ day, that is, day of initiation of FSH treatment was higher in T-I than T-II and control groups. The mean concentration of IGF-I further increased continuously from 11^th^ day to 21^st^ day in the control group (Table-3). The concentration of IGF-I slightly decreased on the 13^th^ day in T-I group and 15^th^ day in T-II group, then increased continuously up to a 21^st^ day. The mean IGF-I concentration started declining following PGF2α injection on 11^th^ day in T-I group and reached lowest on the day 13 i.e., day of superovulatory estrus which differ non-significantly (p>0.05) within groups.

**Table-3:** Serum IGF-I concentration (ng/ml) of different groups on different days of estrous cycle/superovulatory treatment of Sahiwal donors.

Days of the estrous cycle	Groups		

	Control (n=6)	T-I (n=6, insulin at 0.25 IU/kg b. wt. S/C)	T-II (n=6, IGF-I at 10 µg total dose per day S/C)
Day 5	52.14±3.19^aA^	95.86±8.32^aB^	80.3±3.48^baB^
Day 7	74.62±6.58^baA^	104.7±9.10^baB^	78.65±2.80^baA^
Day 9	79.40±6.09^baA^	127.49±5.91^cbaB^	86.26±7.25^baA^
Day 11	80.42±9.82^baA^	130.16±2.26^cbaB^	94.35±2.09^baA^
Day 13	106.00±14.15^b^	121.57±11.47^cba^	111.82±11.70^b^
Day 15	107.15±11.24^bB^	138.15±5.25^cbaC^	74.28±3.97^aA^
Day 17	118.65±16.11^b^	140.02±4.41^cb^	95.92±12.57^ba^
Day 19	119.45±12.30^bBA^	147.25±14.97^cB^	97.61±7.10^baA^
Day 21 or DER	125.8±14.87^b^	151.02±12.88^c^	112.24±8.76^b^

Means bearing different superscripts differed significantly (p<0.05) within the groups (a, b, c, d) and between the groups (A, B). IGF-I=Insulin-like growth factor-1

## Discussion

The mean concentration of serum progesterone on day 5 of estrous cycle is in agreement with Pandey *et al*. [10], who reported values of progesterone <2 ng/ml on this day. Its concentration on the 9^th^ day differs non-significantly but higher in T-I and T-II than control group. These findings are in agreement with the reports of Shukla *et al*. [11] and Gupta *et al*. [12], who reported that exogenous insulin is luteotrophic in nature which increases the serum concentration of progesterone. The higher progesterone levels at second follicular wave lead to more follicular recruitment during superovulatory treatment, which yields more embryos at the day of recovery [13]. The population of medium-sized antral follicles has been reported to be more during 9-13 days of cycle or during CL dominance or at a higher level of progesterone. Thus, follicles recruited more if superovulatory treatment started at a higher level of progesterone. The levels of progesterone remained constant at days 9^th^ and 11^th^ as functional CL was present on the ovary of each cow. The progesterone declined significantly following PGF2α injection in all the groups and reached its basal level on the day of superovulatory estrus (<1 ng/ml). Similar finding was reported by Siddiqui *et al*. [14]. At this time, progesterone level was the lowest of all stages due to induced luteolysis. The mean progesterone concentration increased in all groups following superovulatory estrous to day of embryo recovery and the peak value reached on the day of embryo recovery. The peak value on the day of embryo recovery in T-I group might be due to more number of CL formed after ovulations. These findings are in agreement with the many workers [15,16], who reported that progesterone concentration on days 3-4 post superovulatory estrus up to the day of embryo recovery was higher with the more numbers of CL. The value of progesterone on the day of embryo recovery in this study was much higher than that reported by Siddiqui *et al*. [14] in Sahiwal cows and heifers.

The mean concentration of serum insulin increased in all groups, but on days 7^th^ and 9^th^ difference was significantly higher between control and T-I groups. These findings are in agreement with the reports of Selvaraju *et al*. [17] in repeat breeding cows and Souza *et al*. [18] in goats. It is found that the administration of insulin significantly increased the insulin levels in goats [18] during superovulation. They also found that when insulin was injected before mating, a strong relationship between ovulations and number of transferable embryos was found. However, it has been shown that feeding regimens which increase insulin (hyperinsulinemia) stimulate follicular growth and increase follicular recruitment in heifers [19] and mares [20]. The increase in insulin and IGF-1 levels had a stimulatory effect on follicular growth (small follicle) before superovulation [21]. Insulin regulates CL function, through glucose availability and hormone production in cattle [22].

Our findings on the 5^th^ and 7^th^ days of treatments differ significantly (p<0.05) between groups which are in agreement with the reports of Yelich *et al*. [23] in cow and Salazar-Ortiz *et al*. [20] in mare, who found that the concentrations of IGF-I in plasma are positively associated with body condition and nutrient intake of animals. Obese cows showed higher concentrations of IGF1 in plasma compared with lean cows [24]. Salazar-Ortiz *et al*. [20] also found that the number of follicles appeared to be significantly higher in the well-fed group mares as compared to restricted group. Our findings are also in agreement with the report of Velazquez *et al*. [24], who found that intraovarian application of IGF1 did not affect plasma IGF-1 concentrations in treated and control cow. The levels of IGF-I on the day of embryo recovery were far below as recorded by Velazquez *et al*. [25] in superovulated heifers and cows. In humans, serum concentrations of IGF-I did not allow the prediction of the ovarian response with regard to numbers of oocytes retrieved after superovulation [26]. However, other studies have indicated that plasma [27] concentrations of IGF-I increase during superovulatory treatments. The receptors for IGF-1 are present on uterus and embryo. It was also found that the polymorphism in IGF1R is associated with superovulation traits and indicated that the IGFIR gene can be used as a potential marker for donor selection in cattle [28]. Hence, the growth and development of embryos are affected by serum/plasma IGF-1 concentration.

## Conclusion

Insulin administration during mid-luteal phase may be helpful in follicular and embryonic development through modulation of progesterone, insulin, and IGF-I in indigenous (*B. indicus*) Sahiwal embryo donor cows.

## Authors’ Contributions

The present study was part of SKS’s Ph.D. thesis research. The work was designed by SP and HPG. SP and HPG guided, designed the research work, and drafted the final manuscript. The laboratory work was performed by SKS and SP. All authors read and approved the final manuscript.
